# Technical efficiency of public district hospitals and health centres in Ghana: a pilot study

**DOI:** 10.1186/1478-7547-3-9

**Published:** 2005-09-27

**Authors:** Daniel Osei, Selassi d'Almeida, Melvill O George, Joses M Kirigia, Ayayi Omar Mensah, Lenity H Kainyu

**Affiliations:** 1 Planning and Budget Unit, PPME, Ghana Health Service, Accra, Ghana; 2 WHO Country Office, Accra, Ghana; 3 World Health Organization, Regional Office for Africa, Brazzaville, Congo; 4 Department of Health Sciences, School of Public Health, Kenyatta University, Nairobi, Kenya

## Abstract

**Background:**

The Government of Ghana has been implementing various health sector reforms (e.g. user fees in public health facilities, decentralization, sector-wide approaches to donor coordination) in a bid to improve efficiency in health care. However, to date, except for the pilot study reported in this paper, no attempt has been made to make an estimate of the efficiency of hospitals and/or health centres in Ghana. The objectives of this study, based on data collected in 2000, were: (i) to estimate the relative technical efficiency (TE) and scale efficiency (SE) of a sample of public hospitals and health centres in Ghana; and (ii) to demonstrate policy implications for health sector policy-makers.

**Methods:**

The Data Envelopment Analysis (DEA) approach was used to estimate the efficiency of 17 district hospitals and 17 health centres. This was an exploratory study.

**Results:**

Eight (47%) hospitals were technically inefficient, with an average TE score of 61% and a standard deviation (STD) of 12%. Ten (59%) hospitals were scale inefficient, manifesting an average SE of 81% (STD = 25%). Out of the 17 health centres, 3 (18%) were technically inefficient, with a mean TE score of 49% (STD = 27%). Eight health centres (47%) were scale inefficient, with an average SE score of 84% (STD = 16%).

**Conclusion:**

This pilot study demonstrated to policy-makers the versatility of DEA in measuring inefficiencies among individual facilities and inputs. There is a need for the Planning and Budgeting Unit of the Ghana Health Services to continually monitor the productivity growth, allocative efficiency and technical efficiency of all its health facilities (hospitals and health centres) in the course of the implementation of health sector reforms.

## Background

The strategic health objectives of *Vision 2020 *in Ghana envisage: a significant reduction in the rates of infant, child and maternal mortality; effective control of the risk factors that expose individuals to major communicable diseases; increased access to health services, especially in rural areas; establishment of a health system effectively reoriented toward delivery of public health services; and effective and efficient management of the health system [1].

The Ministry of Health, following the thrust of *Vision 2020*, developed its current policy and strategy guidelines in 1995 in the Medium-Term Health Strategy (MTHS) document [2]. The five main objectives of the MTHS are: improving access to health services; improving quality of care; improving efficiency; fostering partnership between private and public health service-providers; and improving financing of health services.

Subsequently, the first [3] and second [4] Health Sector Five-Year Programme of Work were developed to enable the country attain the MTHS objectives. One of the five underlying objectives of the two programmes of work is "improved efficiency in health services delivery". Furthermore, the Ghana Poverty Reduction Strategy 2002–2004 [5] also highlights "enhancing efficiency in health service delivery" as one of the three priority health sector-related interventions. Thus, efficiency concerns are deeply embedded in the national *Vision 2020*, national poverty reduction strategy, health policy and programme of work.

In a bid to improve the efficiency of health services delivery, the Ministry of Health is implementing the following health sector reforms: separation of functions between the Ministry of Health (policy formulation, planning, donor coordination and resource mobilization) and the Ghana Health Services (responsible for service delivery); autonomy of tertiary hospitals; decentralized planning and budgeting systems, strengthening of financial management and performance monitoring system, and investing in overall management development capacity within the sector; sector-wide approach (SWAP); and strengthening of existing regulatory bodies and laws [6,7].

Since 1978, Data Envelopment Analysis (DEA) has been extensively used in the Americas [8-10], Western Europe [11-17] and Asia [18,19] to shed light on the efficiency of various aspects of national health systems. In Africa, the application of DEA in the health sector has been quite limited. So far, the approach has been applied to health facilities in only three countries, i.e. South Africa [20-22], Kenya [23,24] and Zambia [25]. Yet, the assessment of the efficiency ought to be more prevalent in low-income countries like Ghana in order to optimise health benefits from the available meagre health sector resources.

In Ghana, prior to the current study, no attempt had been made to estimate the efficiency of health care facilities, using either parametric (econometric) or non-parametric methods. The Planning Unit in the Ministry of Health (with support from the World Health Organization) decided to undertake a limited pilot study to demonstrate to policy-makers the potential usefulness of DEA in the pursuit of health sector efficiency objectives. Once policy-makers were adequately sensitised, hopefully, a national efficiency study would be conducted among all health centres and district, regional and tertiary hospitals.

The objectives of the exploratory study reported in this paper were: (i) to estimate the relative technical efficiency of a sample of public hospitals and health centres in Ghana; and (ii) to demonstrate policy implications for health sector policy-makers.

## Methods

### Study area

Ghana is situated on the west coast of Africa. It is divided into ten administrative regions (i.e. Upper East, Upper West, Northern, Brong Ahafo, Ashanti, Volta, Eastern, Greater Accra, Central and Western) and 110 districts. The organisation of health services more or less mimics the administrative structure.

The country's health services are organised at the following levels [2]:

a) *Community: *Delivered through outreach programmes, resident or itinerant herbalists, traditional birth attendants and/or retail drug peddlers.

b) *Sub-district: *A health centre services a geographical area with 15 000 to 30 000 population. It provides basic curative care, disease prevention services and maternity services (primary health care). A health centre constitutes an essential component of the close-to-client health services.

c) *District: *A district hospital provides support to sub-districts in disease prevention and control, health promotion and public health education; referral outpatient and inpatient care, training and supervision of health centres; maternity services, especially the management of complications and emergencies and surgical contraception.

d) *Regional: *A regional hospital provides specialised clinical and diagnostic care; management of high-risk pregnancies and complications of pregnancy; technical and logistical back up for epidemiological surveillance; and research and training.

e) *Tertiary: *At the apex of the referral system, there are two government-owned teaching hospitals that offer specialised services, undertake research, and provide undergraduate and postgraduate training in health and allied areas.

f) *National (i.e. Ministry of Health headquarters): *The national level is responsible for the development of national health policy and for providing strategic directions for service delivery as well as coordination and monitoring.

Ghana's population of 19.7 million is served by a total of 2189 health facilities, of which 952 are government owned, 181 are owned by religious organisations, 75 are quasi-government and 980 belong to the private sector. Out of the total number of health facilities, 2 are teaching hospitals, 9 regional hospitals, 91 district hospitals, 124 other hospitals, 558 health centres, 1085 clinics and 320 are maternity homes. All these health facilities are serviced by 1294 doctors, 29 dentists, 207 pharmacists and 326 medical assistants, along with other paramedical and support staff [7].

The country spends a total of US$ 252 million (4.2% of the GDP of US$ 6 billion) annually on health. About 53.5% of this expenditure is incurred by the government and 46.5% by the households through out-of-pocket expenses. The total per capita expenditure on health at an average exchange rate is US$ 11 [26].

The life expectancy at birth in Ghana is 57.4 years. The infant and under-5-years mortality rates per 1000 live births are 57 and 97 respectively [26]. The probability of dying (per 1000 live births) between ages 15 and 59 years is 303. The maternal mortality per 100 000 live births is 214. Nearly 72% of the population has access to improved sanitation, while 73% has access to an improved water supply source [27].

The question is whether the people of Ghana are deriving maximum health care benefits from the aforementioned investments in health sector, especially from hospitals and health centres which consume over 75% of both the recurrent and capital budgets of the Ministry of Health. The next sub-section presents a DEA conceptual framework, which is used to shed light on this issue.

### DEA conceptual framework

In the production process, hospitals and health centres turn *inputs *(factors of production) into *outputs *(health services). We can divide the inputs into broad categories of labour, materials and capital, each of which will often include more narrow sub-divisions. Labour inputs include skilled health personnel (doctors, nurses, paramedics, support staff) and unskilled workers (drivers, watchmen, gardeners, ward attendants, cooks, etc.), as well as the entrepreneurial efforts of managers of health facilities. Materials include pharmaceuticals, non-pharmaceutical supplies and any other goods that health facilities require to produce health care. Capital includes buildings, medical equipment, vehicles and beds.

**Figure 1:**
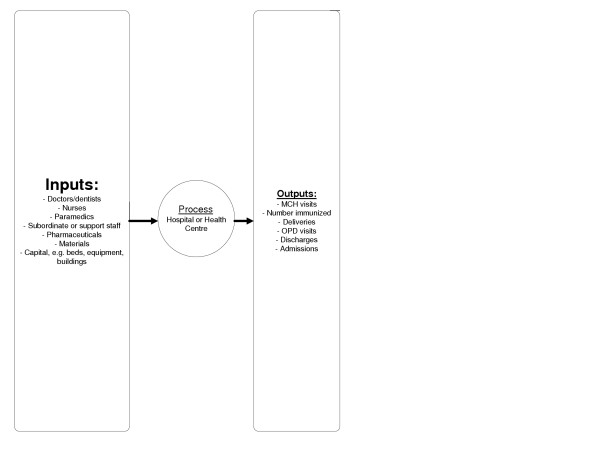
Relationship between inputs and the production process and resulting outputs.

The relationship between inputs and the production process and resulting outputs is described in Figure 1. It is clear that hospitals and health centres employ multiple inputs to produce multiple outputs. We used DEA approach since it allows the measurement of relative efficiency when decision-making units (in this case hospitals/health centres) have multiple inputs and multiple outputs.

DEA is a linear programming methodology for evaluating relative efficiency of each production unit among a set of fairly homogeneous decision-making units (DMUs), e.g. district hospitals, health centres, etc. It sketches a production possibilities frontier (data envelop or efficient frontier) using combinations of inputs and outputs from best performing health facilities. Health facilities that compose the "best practice frontier" are assigned an efficiency score of one (or 100%) and are deemed technically efficient compared to their peers. The efficiency of the health facilities below the efficiency frontier is measured in terms of their distance from the frontier. The inefficient health facilities are assigned a score between one and zero. The larger the score the more efficient a health facility is.

Since hospitals and health centres employ multiple inputs to produce multiple outputs, their individual technical efficiency can be defined as [28]:



The technically inefficient health facility uses more weighted inputs per weighted output, or produce less weighted output per weighted input than those health facilities on the "best practice frontier".

Algebraically, technical efficiency score of each hospital and health centre in the sample were obtained by solving the models (1) and (2) (See Table 8) [29].

**Table 8:** 

**Model 1. DEA weights model, input-oriented, constant returns to scale (CRS)**	**Model 2. DEA weights model, input-oriented, variable returns to scale (VRS)**
	

Where:

*y*_*rj *_= the amount of output *r *produced by hospital or health centre j,

*x*_*ij *_= the amount of input *i *used by hospital or health centre j,

*u*_*r *_= the weight given to output *r*, (r = 1,..., t and t is the number of outputs),

*v*_*i *_= the weight given to input *i*, (i = 1, ..., m and m is the number of inputs),

*n *= the number of hospitals or health centres,

j_0 _= the hospital or health centre under assessment.

We need to explain what we mean by constant returns to scale and variable returns to scale. Returns to scale refers to the changes in output as all inputs change by the same proportion. For instance, suppose that for a specific hospital (or health centre) j we start from an initial level of inputs (doctors = D, Other technical staff = T, Subordinate staff = S, Beds = B) and output (Q)

*Q*_*o *_= *f*(*D*,*T*,*S*,*B*)

and we increase all the factors by the same proportion φ. We will obviously obtain a new level of output Q*, higher than the original level *Q*_0_,

Q* = *f*(φ*D*,φ*T*,φ*S*,φ*B*)

If Q* increases by: (i) the same proportion φ as the inputs, we say that there are constant returns to scale (CRS); (ii) less than proportionally with the increase in inputs, we have decreasing returns to scale (DRS); (iii) more than proportionally with the increase in the inputs, we increasing returns to scale (IRS).

Those hospitals and health centres manifesting CRS can be said to be operating at their most productive scale sizes. In order to operate at the most productive scale size, a health facility displaying DRS should scale down both outputs and inputs. If a health facility is exhibiting IRS, it should expand both outputs and inputs in order to become scale efficient [8].

We have illustrated below how DEA works using hypothetical hospitals.

### Illustration of the DEA analysis

Lets assume that a hypothetical country called Nkrumah has 9 district hospitals. Each hospital produces two outputs (i.e. outpatient department visits (OPVisits) and inpatient admissions (Admissions)) from a single input of technical staff. The number of staff employed, Opvisits, Admissions, ratios of Opvisits to staff, and Admissions to staff are contained in the Table 1.

**Table 1:** Illustration of DEA analysis using a hypothetical example of nine hospitals

**DMUs**	**OPVisits (A)**	**Admissions (B)**	**Staff (C)**	**OPvisits/staff D = (A/C)**	**Admissions/staff E = (B/C)**
Anim	7020	5451	102	69	53
Akoa	20566	7610	92	224	83
Addai	25200	7148	143	176	50
Mensa	33568	7958	96	350	83
Kofi	17406	2429	95	183	26
Gyau	10573	8094	117	90	69
Anani	10500	8944	115	91	78
Amoi	20421	10969	117	175	94
Asamoa	20647	8619	46	449	187

The efficiency of each hospital in producing the two outputs were estimated by dividing each of their outputs by their input and seeing which hospital(s) have the highest ratios. The results are contained in the last two columns of Table 1. The higher the ratio of an output to input the more efficient a hospital is in producing that output. In this example, Asamoa hospital had the highest number of Opvisits per staff (449) and Admissions (187) for each member of technical staff employed.

**Figure 2:**
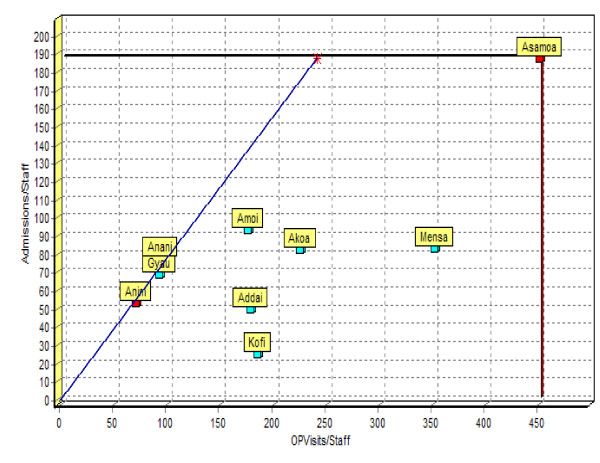
Production possibilities frontier graph.

By plotting Opvisits/staff against Admissions/staff for the nine hospitals we derive the production possibilities frontier graph contained in Figure 2.

The diagram shows the efficiency frontier, which is the fundamental concept of DEA. The straight lines from Asamoa hospital to the Y axis (labelled Admissions/staff) and from Asamoa to the X axis (labelled Opvisits/staff) represent the efficient frontier. The efficiency frontier, derived from the most efficient hospital(s) (i.e. Asamoa hospital in our example) in the dataset/sample, represents a standard of technically best performance that can be achieved from available input and technology endowment. Consequently, it is used as a threshold against which to measure the performance of all other hospitals.

The efficiency frontier 'envelops' the inefficient hospitals within it and clearly shows the relative efficiency of each hospital. A hospital like Asamoa, which located on the frontier, is considered 100% technically efficient. Any hospital like Mensa, Amoi, Akoa, Anani, Kofi, Addai, Gyau and Anim that is below the production possibilities frontier is relatively less efficient and is given a technical efficiency rating of less than 100%.

Anim hospital, for instance, could become efficient if it increased its outputs, in the same proportions, while holding its input constant, i.e. assuming an output-orientated model/situation. Instead, it could become efficient by reducing its input while keeping its outputs the same, i.e. assuming an input-orientated scenario. Its technical efficiency is calculated by the ratio of its distance from the origin over the distance from the origin to the point of intersection on the production possibilities frontier or the efficiency frontier. This gives Anim hospital a technical efficiency score of 28.52%. Likewise, Mensa hospital is 77.90% as efficient as Asamoa hospital (i.e. the best practice hospital), Amoi hospital is 50.04%, Akoa hospital is 49.80%, Anani hospital is 41.51%, Kofi hospital is 40.82%, Addai hospital is 39.26%, and Gyau hospital is 36.92%. These scores were estimated assuming constant returns to scale (CRS).

However, often health services production process are not linear, and thus it may be more appropriate to assume variable returns to scale (VRS). Thus, we estimated the DEA model assuming VRS, the efficiency scores for various hospitals were as follows: Asamoa = 100%, Amoi = 100%, Mensa = 100%, Akoa = 50%, Anani = 48.54%, Kofi = 48.42%, Anim = 45.10%, Addai = 44.49% and Gyau = 39.32%. This finding implies that if Akoa, Anani, Kofi, Anim, Addai and Gyau hospitals were to operate efficiently, they are capable of producing their current output levels with 50%, 51.5%, 51.58%, 54.9%, 55.51% and 60.68% less inputs than they are currently using. Whereas in the CRS model only Asamoa hospital had a 100% efficiency score, in the VRS model three hospitals (Asamoa, Amoi, Mensa) achieved a pure technical efficiency score of 100%.

The impact of hospital scale/size on their technical efficiency was evaluated using a three-step process. First, the model was estimated assuming CRS. Second, the model was run assuming VRS. Third, scale efficiency was obtained by dividing each hospital's CRS technical efficiency score by its VRS technical efficiency score. Akoa and Asamoa hospitals has scale efficiency score of 100%, implying they had an optimal size. Gyau scored 94%, Addai scored 88%, Anani scored 86%, Kofi scored 84%, Mensa scored 78%, Anim scored 63% and Amoi scored 50%. These seven hospitals were scale inefficient since they were not operating at their most productive size for their observed input mix. It is important to mention that DEA is only an exploratory tool for efficiency measurement, and indicates directions for further investigations into how to improve/enhance efficiency.

### Input and output orientation

#### Input Orientation for hospitals

In the hospital analysis the input orientation assumed that these facilities had limited control over the volume of their outputs. There was no linkage between staff earnings and output; thus, there was no incentive for inducing demand for health services. Otherwise, in Ghana hospital management has got greater control over the use of inputs. Thus, an input-oriented DEA model was used for hospital analysis.

#### Output orientation for health centres

On the other hand, output orientation was assumed for health centres. The management of health centres has no control over inputs, especially it's staffing. However, given their primary health care orientation, with a strong bias towards health promotion and disease prevention, they can influence a great number of people seeking, for example, antenatal and postnatal care, family planning services, birthing services, immunisations and health education, through their public health outreach work among communities. Thus, the output-oriented DEA model was used for the health centre analysis.

### Strengths and weaknesses of DEA

#### Strengths of DEA

We chose to employ DEA approach to estimate technical efficiency of individual hospitals and health centres because of its unique strengthens: (i) it can handle multiple input and multiple output models/scenarios typical of hospitals and health centres; (ii) it does not require an assumption of a functional form relating inputs to output (as regression methods do); (iii) health facilities are directly compared against a peer or combination of peers; (iv) inputs and outputs can be very different units; (v) it does not require information on prices of inputs and outputs [22,30].

#### Weaknesses of DEA

Even though we chose to use DEA, we were fully aware that it has two main limitations. Firstly, it attributes any deviation from the "best practice frontier" to inefficiency, while some could be due to statistical noise, e.g. epidemics or measurement errors. Secondly, given that DEA is deterministic/nonparametric technique, it is difficult to conduct statistical tests of hypotheses concerning the inefficiency and the structure of the production function [22,31,32].

### Variables

The hospital DEA model had a total of 7 variables, including 3 outputs and 4 inputs. The three outputs for each individual hospital were: (i) the number of maternal and child care (MCH) (i.e. antenatal care, postnatal care, family planning, tetanus toxoid, child immunisation and growth monitoring); (ii) the number of child deliveries; and (iii) the number of patients discharged (not including deaths). The four inputs included: (i) number of medical officers; (ii) the number of technical officers (including medical assistants, nurses and paramedical staff); (iii) the support or subordinate staff (including orderlies, ward assistants, cleaners, drivers, gardeners, watchmen, etc.); and (iv) the number of hospital beds.

On the other hand, the health centre DEA model was estimated with a total of 6 variables: 4 outputs and 2 inputs. The four outputs for each individual health centre were: (i) the number of child deliveries; (ii) the number of fully immunised children under the age of 5 years; (iii) the number of other maternal (i.e. antenatal care, postnatal care and family planning services) and childcare (nutritional/child growth monitoring) visits; and (iv) the number of outpatient curative visits. The two inputs were: (i) the number of technical staff (this included medical assistants, nurses and paramedical staff); and (ii) the number of support or subordinate staff (including cleaners, drivers, gardeners, watchmen and others).

The choice of inputs and outputs for the DEA analysis was guided in part by the previous DEA health care studies in the African Region and availability of data.

### Data

The data used in this study are for 2000. In order to have a feel of the usefulness of DEA in the measurement technical efficiency of hospitals and health centres, the policy-makers instructed the Planning and Budget Unit (PBU) to draw a pilot sample of 21 hospitals and 21 health centres. PBU decided to use simple random sampling technique to draw the two samples. Data were collected from a random sample of 21 public health centres using a WHO African Regional Office (WHO/AFRO) efficiency questionnaire for primary health care facilities [32]. However, information on health personnel in four health centres was missing; thus, they were left out of the analysis. Data were also collected from a random sample of 21 district hospitals; however, information on inputs and outputs from only 17 hospitals was included in the analysis. Data on hospitals were collected using a WHO/AFRO efficiency questionnaire for hospitals [33]. The data were analysed using the DEAP software developed by Professor Tim Coelli [31].

## Results

### Hospitals analysis

In 2000, all 17 hospitals in the sample produced a total of 200 589 maternal and child health (MCH) visits, 24 152 deliveries and 69 361 discharges. Those outputs were produced employing a total of 55 medical officers/dentists, 1345 technical staff, 721 subordinate staff and 1543 beds. Table 2 presents the means and standard deviations for input and output variables of the 17 district hospitals.

**Table 2:** Means and standard deviations for public hospitals inputs and outputs

**Variable**	**Mean**	**Standard deviation**
Outputs: Maternal and child health care visits	11799	9516
Deliveries	1421	1186
Inpatient discharges	4080	2274
Inputs: Doctors/dentists	3	3
Technical staff (including nurses)	79	34
Subordinate staff	42	27
Beds	91	43

The VRS model technical and scale efficiency scores for individual hospitals are contained in Table 3. Of the 17 hospitals, 9 (53%) were technically efficient since they had a relative technical efficiency (TE) score of 100%. The remaining 8 (47%) had a TE score of less than 100%, which means that they were technically inefficient. The TE score among the latter facilities ranged from 74% in Atua hospital to 43% in Winneba hospital. This finding implies that Atua and Winneba hospitals could potentially reduce their current input endowments by 26% and 57% while leaving their output levels unchanged. The average TE score among the inefficient hospitals was 61% (standard deviation = 12%), which means that these hospitals could, on average, produce their current levels of output with 39% less inputs than they were currently using.

**Table 3:** Technical and scale efficiency scores for district hospitals

**DMU (Hospitals)**	**Technical efficiency (%)**	**Scale efficiency (%)**
Swendru	100	100
Half Asin	100	100
Turkwa	100	100
Kwesiminstmu	100	100
Yendi	100	100
Takoradi	100	100
St Francis Xavier	100	100
West End	100	91.6
Walewale	100	43.9
Atua	74.4	99.8
Tetteh Quarshie	73.6	99.9
Cape Coast	68.6	93.2
Akuse	66.3	43.9
Axim	57.4	50.9
Suhum	56.5	98.0
Akim	46.0	99.6
Winneba	42.7	93.9

Seven (41%) of the hospitals had a scale efficiency (SE) of 100%, which means that they had the most productive size for that particular input-output mix. The remaining 10 (59%) hospitals had a SE of less than 100% and as such they were scale inefficient. The average SE among the inefficient hospitals was 81% (standard deviation = 25%), meaning that, on average, the scale inefficient hospitals could reduce their size by 19% without affecting their current output levels.

All the seven scale-efficient hospitals displayed constant returns to scale (CRS), implying thereby that they were operating at their most productive scale sizes. Eight of the 10 scale-inefficient hospitals had increasing returns to scale (IRS) while one of the hospitals revealed decreasing returns to scale (DRS). In order to operate at the most productive scale size (MPSS), a hospital exhibiting DRS should scale down both its outputs and inputs. Similarly, if a hospital is displaying IRS, it should expand both its outputs and inputs.

### Health centre analysis

The total output of all health centres in the sample combined was as follows: (i) 67 739 MCH visits; (ii) 4541 deliveries; (iii) 28 909 fully immunised children; and (iv) 81 665 outpatient curative visits. The total input endowment of all health centres consisted of 181 technical staff and 87 subordinate staff. Table 4 presents the means and standard deviations for input and output variables of 17 health centres.

**Table 4:** Means and standard deviations for public health centres inputs and outputs

**Variables**	**Mean**	**Standard deviation**
Outputs: Maternal and child health care visits	3985	2579
Deliveries	267	207
Fully-immunized children	1701	1526
OPD curative visits	4804	5475
Inputs: Medical assistants/nurses/other technical staff	11	5
Subordinate staff	5	3

The VRS technical and scale efficiency scores for individual health centres are given in Table 5. Out of the 17 health centres, 14 (82%) were technically efficient since they had a relative technical efficiency (TE) score of 100%. The remaining 3 (18%) had a TE score of less than 100% and thus were deemed to be technically inefficient. The TE score among the latter facilities varied from about 80% at Daboase and 42% at Tikobo to 27% at the Abomoso health centre. This means that Daboase, Tikobo and Abomoso could potentially produce 20%, 58% and 73% more outputs respectively using their current input endowment if they were to operate efficiently.

**Table 5:** Technical and scale efficiency scores for public health centres

**DMU (Health centres)**	**Technical efficiency (%)**	**Scale efficiency (%)**
Diare	100	100
Nafong	100	100
Anyingse	100	100
Anyinam	100	100
Akroso	100	100
Elubo	100	100
Adisadel	100	100
Nkwanyawum	100	100
Adukrom	100	100
Osino	100	98.3
Fanti Nyankomasia	100	90.1
Okrakwadwo	100	88.7
Ewim	100	69.5
Savelugu	100	67.6
Daboase	79.7	58.5
Tikobo	42.0	96.6
Abomoso	26.6	99.9

On the other hand, 9 (53%) of the health centres were scale efficient because they had a relative scale efficiency (SE) score of 100%. The remaining 8 health centres (47%) had a SE of less than 100%, and as such they were scale inefficient. The average SE among the inefficient health centres was 84% (standard deviation = 16%). This implies that, on average, the scale inefficient health centres could produce their current output levels with 17% less capacity than they were actually using.

All the 9 scale-efficient health centres exhibited constant returns to scale (CRS). Except for Abomoso, all the other 7 scale-inefficient health centres manifested decreasing returns to scale (DRS).

## Discussion

### Hospitals analysis

#### Key Findings

Forty-seven per cent of the hospitals in the sample were technically inefficient and 59% of them were scale inefficient. A similar study among 55 public hospitals in Kwazulu-Natal province in South Africa found 40% of the hospitals to be technically inefficient and 42% to be scale inefficient [20,22]. Another DEA analysis of 54 public hospitals in Kenya revealed that 26% of them were technically inefficient while about 30% were scale inefficient [23]. Masiye *et al. *[25] undertook DEA among 20 hospitals in Zambia and found 75% of them to be technically inefficient. Thus, the available evidence indicates that although there is significant technical inefficiency among health facilities in Ghana, Kenya, South Africa and Zambia, the magnitude of inefficiency does vary.

Table 6 shows the total output increases and/or input reductions needed to make inefficient district public hospitals efficient. The inefficient hospitals could be technically efficient if they were to increase their output levels by 25% more MCH visits, 12% more deliveries and 1% more discharges, while holding their current input endowment constant. Alternatively, the inefficient hospitals could become technically efficient if they were to reduce their current number of medical officers/dentists by 44%, technical staff by 22%, and subordinate staff by 28% and beds by 29% while holding the output constant.

**Table 6:** Total output increases and/or input reductions needed to make inefficient district public hospitals efficient

**Variables**	**Radial movement (A)**	**Slack movement (B)**	**Total Value (C = A+B)**
Outputs: Maternal and child Health care visits	0	50845	50845
Deliveries	0	2865	2865
Inpatient discharges	0	808	808
Inputs: Doctors/dentists	13	11	24
Technical staff (including nurses)	292	0	292
Subordinate staff	173	27	200
Beds	397	51	448

#### Policy implications for hospitals

In regard to the excess resources, which were wasted and not utilized in the production of hospital outputs, decision-makers in the Ghana Ministry of Health have a number of policy options available to them. These are as follows:

a) Do nothing and continue with the wasteful situation as it exists. However, judging from the strategic and policy documents produced by the Ministry of Health, it is clear that this option is considered unacceptable by its policy-makers.

b) Option related to excess medical officers/dentists and other technical staff: In our opinion, given the need for strengthening health services at sub-district and community levels, it would not be rational to offer any category of technical staff the option of early retirement. Instead, excess medical officers/dentists and other technical staff should be transferred to health centres to provide primary health care. We believe that this would increase health coverage and quality of service provided at sub-district and community levels.

c) Options related to excess subordinate staff include: (i) Offering employees early retirement with severance pay plans (i.e. voluntary retirement); (ii) forced retrenchment with severance package; and (iii) transfer of excess labour force to under-staffed primary health care facilities.

d) Options related to excess beds and space include: (i) Convert the space excess beds occupy to provide outpatients secondary prevention services; (ii) rent excess beds and space to private medical practitioners if there is a demand for them; and (iii) sell excess beds and the space they occupy and use the money thus realised to improve the quality of hospital care.

e) Closure of some hospitals: Holding equity and political concerns constant, in principle, health policy-makers could opt to close down those hospitals with efficiency score below a certain threshold. However, in reality, members of parliament representing the concerned constituencies might be opposed to such an option due to potential political fallout.

f) Conversion of specific hospitals into health centres: This option would entail downsizing both the services provided and staff composition and their numbers. If this option were to be pursued, there would be need for working out details of the conversion process.

Implementation of any of the aforementioned options will need to be preceded by more detailed studies into the determinants of inefficiencies.

### Health centres analysis

#### Key findings

In the Ghana pilot study reported in the paper, 18% of the health centres were technically inefficient and 47% were scale inefficient. A DEA study of 155 primary health care clinics in Kwazulu-Natal province in South Africa found 70% of them to be technically inefficient while 84% manifested some scale inefficiency [21]. A similar study of 32 public health centres in Kenya revealed that 56% of them were technically inefficient while 41% were scale inefficient [24].

Table 7 presents the total output increases and/or input reductions needed to make inefficient public health centres efficient. In order to become efficient, health centres will need to expand their: (i) maternal and child health visits by 9%; (ii) deliveries by 11%; (iii) fully-immunised children by 9%; and (iv) outpatient curative visits by 6%. If the excess inputs in district hospitals were to be transferred to primary health care facilities, health centres would potentially be able to increase their outputs by even larger magnitudes than those indicated above.

**Table 7:** Total output increases and/or input reductions needed to make inefficient public health centres efficient

**Variable**	**Radial movement (A)**	**Slack movement (B)**	**Total movement (C = A+B)**
Outputs: Maternal and child health care visits	5306	1100	6406
Deliveries	507	0	507
Fully-immunized children	2645	74	2719
OPD curative visits	3451	1564	5015
Inputs: Medical assistants/nurses/other technical staff	0	7	7
Subordinate staff	0	1	1

#### Policy implications for health centres

Health centres provide affordable promotive, preventive and basic curative care in localities inhabited mainly by the poor. Their location makes them critically important in the ongoing efforts to scale up pro-poor cost-effective public health interventions geared at achieving the health-related Millennium Development Goals [34] and New Partnership for Africa's Development (NEPAD) health targets [35]. Thus, the importance of these close-to-client health facilities in all efforts to reduce the burden of disease and improve health conditions, especially in rural areas, cannot be overemphasised.

Health promotion strategies and methods may be crucial in inducing the necessary demand for services mentioned above in order to reduce technical inefficiencies in health centres [36]. Health promotion uses approaches/methods such as advocacy (including lobbying), health education, communication for behavioural change, social marketing, social mobilisation, information, education and communication (IEC), legislation and economic and environmental policies to reduce health risks involving health as well as non-health sectors (e.g. agriculture, education, housing, sanitation, trade, transport, water) [37,38].

Given their strategic position amongst communities and closeness to actual and potential clients, health centres make a vital contribution to the development, implementation, monitoring and evaluation of health-promoting initiatives. For example, through the combined use of the aforementioned health promotion strategies and approaches, health centre personnel (with some additional basic health-promotion training) can proactively motivate and persuade households in order to:

• support pregnant women to seek antenatal care, to give birth under the care of skilled birth attendants and seek postnatal care;

• get their children immunised against vaccine-preventable diseases;

• take their children to outpatient departments for integrated management of childhood illnesses (IMCI).

Apart from health promotion, once the Ghana National Health Insurance (GNHI) programme is fully implemented up to the community level, demand for health services is bound to increase due to reduction in financial barriers. The GNHI consists of Social Health Insurance Schemes (District Mutual Health Insurance Schemes and Private Mutual Health Insurance Schemes) and Private Commercial Health Insurance Schemes [39,40].

### Limitations of the study

It could be argued that the objective function of health facilities is to maximise health gains from available resources. And the ideal output indicator would be the one that captures in a sensitive, valid, reliable and culturally acceptable manner changes in both the quantity and quality of the lives of those who interact with hospitals and health centres [20]. However, given the unavailability of data on either Disability-Adjusted Life Expectancy (DALE) [41] or Quality-Adjusted Life Years (QALY) [42,43] gained due to care in each of the facilities in the data set, we opted to use proxies that had been used in similar studies in the past [20-24].

It may be argued that there may be variation in the quality of care provided by different health facilities, e.g. facilities offering higher quality of care may require more personnel time and other inputs than those offering low quality of care. Given the fact that all the hospitals studied were district-level public hospitals, designed and resourced to provide a fairly similar level and mix of care, it is unlikely that there would be any significant variance in the quality of care across these facilities. The health centres studied were also fairly homogeneous in size and mix of services provided [2].

The analysis assumed that the case mix of a specific hospital and its Efficiency Reference Set (ERS) hospitals was similar. We were not able to verify whether that assumption was plausible. However, the fact that the study hospitals were all non-specialist first referral-level hospitals, the above assumption will most likely hold.

Drugs were largely supplied from the Central Medical Stores. However, some health facilities often used their cost-sharing funds to make supplementary acquisitions as and when needed. Unfortunately, data on drug expenditure were not forthcoming in most of the questionnaires; thus, it was decided to drop this variable from the analysis altogether.

Lastly, since the sample for health centres constituted only 3.7% of the total number of public health centres and hospitals formed about 22% of the public district hospitals, the results cannot be generalized to the entire population of health centres and hospitals in Ghana.

### Suggestions for further research

In the light of the challenges of health financing, equity and efficiency (both allocative and technical) confronting the public health sector, there is an urgent need for:

• Conducting technical and allocative efficiency studies in all the public, private-for-profit and religious mission hospitals and health centres with a view to identifying inefficiencies in individual health facilities and their inputs;

• Conducting Malmquist Productivity Index analysis to monitor and evaluate the effects of different health care reforms on productivity growth, technical progress and efficiency change in health facilities over time [44].

## Conclusion

Various governments in Africa have embarked on health sector reforms to improve the performance of their national health systems. Monitoring and evaluating the effects of those reforms on productivity growth, technical progress and efficiency change of fixed health facilities that consume the majority of the recurrent and development budgets of ministries of health is of paramount importance. Our study tried to contribute to establishing baseline technical and scale efficiency information that could be used in monitoring the efficiency effects of future policy changes. We have also briefly described how health promotion strategies and methods could be used to reduce inefficiencies in health centres.

## Competing interests

The author(s) declare that they have no competing interests.

## Authors' contributions

DO, SD and MOG collected the data and participated in analysis and drafting of sections of the document. OM, LHK and DG participated in drafting sections of the manuscript. JMK did literature review and participated in the development of the conceptual framework, data analysis and writing of sections of the document. All the authors read and approved the final manuscript.
